# Load-dependent modulation of non-muscle myosin-2A function by tropomyosin 4.2

**DOI:** 10.1038/srep20554

**Published:** 2016-02-05

**Authors:** Nikolas Hundt, Walter Steffen, Salma Pathan-Chhatbar, Manuel H. Taft, Dietmar J. Manstein

**Affiliations:** 1 Institute for Biophysical Chemistry, Hannover Medical School, Carl-Neuberg-Str. 1, 30625 Hannover, Germany; 2 Molecular and Cell Physiology, Hannover Medical School, Carl-Neuberg-Str. 1, 30625 Hannover, Germany; 3 Research Division for Structural Analysis, Hannover Medical School, Carl-Neuberg-Str. 1, 30625 Hannover, Germany

## Abstract

Tropomyosin isoforms play an important role in the organisation of cytoplasmic actomyosin complexes in regard to function and cellular localisation. In particular, Tpm4.2 is upregulated in rapidly migrating cells and responsible for the specific recruitment of the cytoplasmic class-2 myosin NM-2A to actin filaments during the formation of stress fibres. Here, we investigate how the decoration of F-actin with Tpm4.2 affects the motor properties of NM-2A under conditions of low and high load. In the absence of external forces, decoration of actin filaments with Tpm4.2 does not affect the gated release of ADP from NM-2A and the transition from strong to weak actin-binding states. In the presence of resisting loads, our results reveal a marked increase in the mechanosensitive gating between the leading and trailing myosin head. Thereby, the processive behaviour of NM-2A is enhanced in the presence of resisting loads. The load- and Tpm4.2-induced changes in the functional behaviour of NM-2A are in good agreement with the role of this myosin in the context of stress fibres and the maintenance of cellular tension.

The tropomyosin (Tpm) family consists of more than 40 isoforms generated by alternative splicing from four genes. The great majority of Tpm isoforms are classified as cytoskeletal isoforms^1^. They associate with actin filaments made from cytoskeletal β- or γ-actin and are responsible for the formation and segregation of actin filament populations that display distinct localization patterns, turnover dynamics, and cellular functions (for reviews see^2^,^3^). Compositional variation of cytoplasmic actin-Tpm complexes appears to provide a mechanism for the selective recruitment of myosin isoforms^4^,^5^,^6^. Studies in yeast show that decoration of actin with cytoplasmic Tpm isoforms is required for myosin-5 to show processive behaviour^7^,^8^. The low molecular weight, cytoplasmic isoform Tpm4.2 is produced in a wide range of human cells and tissues. Depending on the cell type, Tpm4.2 appears to play a role in supporting different motile and contractile events. Tpm4.2 is prominent in growth cones of developing neurons, plays an essential role in the formation of a cap over the inner face of the sealing zone in osteoclasts, stabilizes actin-based structures associated with podosomes, and appears to contribute to the formation of focal adhesions and stress fibres in fibroblasts^9^,^10^,^11^. Tpm4.2 production is upregulated as contractile smooth muscle cells dedifferentiate into non-contractile, migrating cell types and some types of cancer cells. In the case of infiltrating ductal breast carcinomas, the aggressiveness of the tumour has been directly linked to Tpm4 expression^12^. The myofibroblastic cells in some inflammatory myofibroblastic tumours contain chromosomal rearrangements involving *TPM4* and *ALK*, which encodes a receptor tyrosine-kinase. While *ALK* expression is normally restricted to neural tissues, the fusion protein is overproduced by the myofibroblastic spindle cells that make up this tumour, which can occur in many different parts of the body^13^.

Mammals produce three distinct filament-forming non-muscle myosin-2 isoforms (NM-2A, NM-2B, NM-2C). The corresponding myosin heavy chains are encoded by three different genes (*MYH9*, *MYH10*, *MYH14*). All three NM-2 isoforms require activation by phosphorylation of their regulatory light chain^14^. However, even in the activated form, they display much slower ATP turnover and motile activity than muscle myosin-2. Activated NM-2 heavy chain dimers assemble into bipolar myosin bundles via their coiled-coil tails. Bipolar NM-2 bundles are responsible for the contraction of antiparallel actin filaments that are found in cytoskeletal structures such as stress fibres and the contractile rings formed during cytokinesis (for reviews see^14^,^15^). Despite several similarities, NM-2 isoforms display distinct enzymatic properties and subcellular localisation^16^,^17^,^18^,^19^,^20^,^21^. The transition from the myosin-ADP-P_i_ state with weak actin-affinity to the myosin-ADP state displaying strong actin-binding was shown to be rate-limiting for NM-2A, while the rate of the subsequent ADP release step is high in comparison with NM-2B^18^,^21^. In the *in vitro* motility assay, NM-2A propels actin filaments 2-3 times faster than NM-2B or NM-2C^17^,^22^. Therefore, NM-2A was classified as a low duty-ratio motor, similar to muscle myosins^18^. However, a later study qualified these results by providing evidence for the force-sensitivity of the ADP release step from double-headed NM-2A^23^.

Four congenital disorders, known as Epstein syndrome, Fechtner syndrome, May-Hegglin anomaly, and Sebastian platelet syndrome, describe different clinical manifestations of mutations that lead to changes within the NM-2A heavy chain. Due to their overlapping nature, they have recently been summarised under the common term MYH9-related diseases (MYH9-RD)^24^. All affected individuals suffer from macrothrombocytopenia and some develop additional clinical manifestations such as hearing loss, renal failure, and formation of presenile cataracts^25^. Individuals with mutations affecting the NM-2A motor domain have a much higher risk for syndromic manifestations than individuals with mutations in the tail region^26^. This observation highlights the importance of specific kinetic properties and the changes in the duty-ratio for disease-related processes, which vary significantly among NM-2 isoforms and are modulated by Tpm-decoration of actin filaments. Therefore, a better understanding of the mechanisms that affect the duty-cycle of physiological NM-2A complexes is of key importance for the rational development and optimisation of small molecule-based therapeutic approaches, aiming to improve the condition of patients suffering from NM-2A-linked congenital disorders.

Here, we provide experimental evidence for the processive stepping of an NM-2A-HMM construct on actin filaments and show how the decoration of actin filaments with Tpm4.2 promotes the processive behaviour of the NM-2A motor in a load-dependent manner.

## Results

### Tpm4.2 facilitates the transition of NM-2A to the strongly actin-binding myosin-ADP state

We produced active, soluble NM-2A motor constructs using the Sf9/baculovirus system. HMM- and S1-like myosin heavy chain fragments were co-produced with human native essential and regulatory light chains MYL6 and MYL12b. Typical yields were 0.8 mg NM-2A-HMM and 9 mg NM-2A-S1 from 2 · 10^9^ cells. The S1 construct is constitutively active, while the HMM construct requires activation by phosphorylation of MYL12b^15^,^27^. Tpm4.2 was produced in *E. coli* with yields of approximately 1.3 mg of homogeneous protein per litre of culture medium (Supplementary Fig. S1).

**Figure 1:**
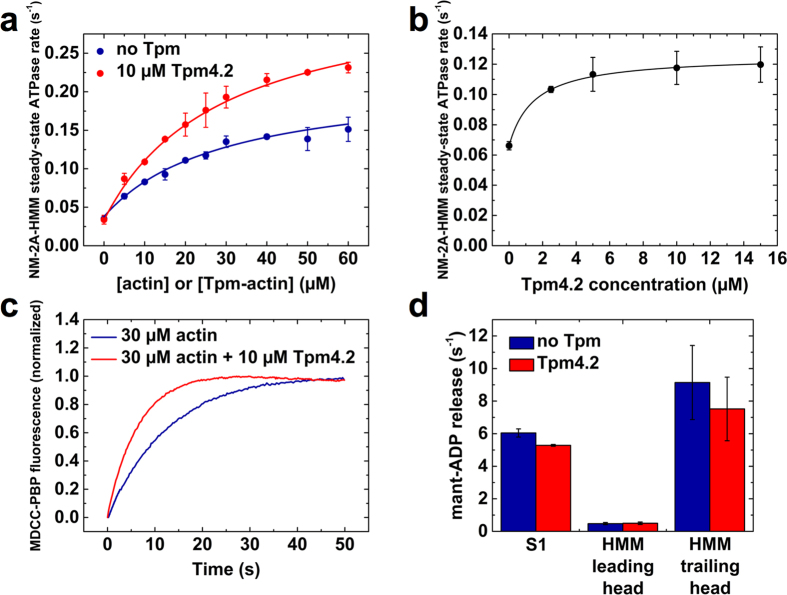
Tpm4.2 facilitates the transition to the strongly-bound actomyosin-ADP state **(a)** The actin-activation of the NM-2A-HMM steady-state ATPase rate is increased by the presence of Tpm4.2. Error bars indicate the standard error of the mean. Data were described with a hyperbolic function. (n = 3) **(b)** The NM-2A-HMM steady-state ATPase rate in the presence of 10 μM F-actin was determined at different Tpm4.2 concentrations. The rate reaches a plateau value at concentrations above 5 μM Tpm4.2. Phenomenologically, the data can be described by a hyperbolic function. **(c)** Phosphate release from NM-2A-S1 is accelerated on Tpm4.2-decorated actin filaments. 2.8 μM NM-2A-S1 was rapidly mixed with 2 μM ATP and incubated for 5 s to form the myosin-ADP-P_i_ complex. It was then mixed with actin or Tpm4.2-actin at a final concentration of 30 μM. Phosphate release was followed by the fluorescence increase of the phosphate sensor MDCC-PBP. **(d)** NM-2A-HMM displays gated ADP release. The preformed complex of actin-NM-2A-S1 with mant-ADP was rapidly mixed with 4 mM ATP and mant-ADP release was followed by the drop in mant fluorescence. The resulting transients were described by a single exponential model (Supplementary Figure S3a and b). The preformed complex of actin-NM-2A-HMM with mant-ADP was rapidly mixed with a large excess of ADP and mant-ADP release was followed by the associated drop in mant fluorescence. The resulting transients were described by a double exponential model (Supplementary Figure S3c and d). The slow phase corresponds to mant-ADP release from the leading head, which experiences resisting strain from the trailing head. The fast phase corresponds to mant-ADP release from the trailing head, which experiences assisting strain from the leading head. Error bars indicate the standard deviation of at least three independent experiments.

To elucidate how the decoration of actin filaments with Tpm4.2 influences their interaction with NM-2A, we measured the actin-activated ATPase activity of NM-2A-HMM during steady-state at varying concentrations of bare and Tpm4.2-decorated F-actin (Fig. 1a). Using bare F-actin, the maximum turnover rate k_cat_ of NM-2A-HMM was 0.22 ± 0.03 s^−1^. In the presence of 10 μM Tpm4.2, k_cat_ increased 1.5-fold to 0.34 ± 0.02 s^−1^. The apparent dissociation equilibrium constant for F-actin binding in the presence of ATP (K_actin_), which is equal to the actin concentration at the half-maximum rate, was unchanged by the presence of Tpm4.2 (see Table 1). Similar results were obtained for NM-2A-S1 (see Supplementary Figure S2 and Table 1). The activating effect on the NM-2A-HMM ATPase rate reached a plateau value at a concentration greater than 10 μM Tpm4.2 (Fig. 1b). It appears likely that saturation is reached at lower concentrations with the N-terminally acetylated form of Tpm4.2^28^.

The rate-limiting step in the ATPase cycle of NM-2A was shown to be the transition from the myosin-ADP-P_i_ state to the strongly actin-bound myosin-ADP state^18^. We wanted to examine whether the increase of the maximum ATPase rate is caused by the acceleration of this step. To facilitate the interpretation of the signals in the stopped-flow experiments, we used single-headed NM-2A-S1. Using the sequential mixing mode of a stopped-flow system, NM-2A was mixed with ATP, aged for 5 s in order to form a myosin-ADP-P_i_ complex, before mixing with bare or Tpm4.2-decorated F-actin. Phosphate release was followed by the fluorescence increase of the phosphate sensor MDCC-PBP (see methods). As shown in Fig. 1c, under the experimental conditions, the dissociation rate constant for the release of phosphate from NM-2A-S1 is more than 2-fold faster with Tpm4.2-decorated actin filaments (0.179 ± 0.012 s^−1^) compared to bare filaments (0.084 ± 0.003 s^−1^). This finding supports the notion that the decoration of F-actin with Tpm4.2 accelerates the transition of the NM-2A motor to the strongly actin-bound states.

ADP release was shown to limit the detachment rate of NM-2A from actin at saturated ATP concentrations^18^. Therefore, a change in the rate of ADP release has a major impact on the fraction of time NM-2A spends in strongly actin-bound states. We measured the ADP release rate from NM-2A-S1 on bare and Tpm4.2-decorated actin filaments by rapidly mixing an actin-NM-2A-S1-mant-ADP complex with a large excess of ATP (4 mM). Under these conditions, the rate of ATP-binding is limited by the dissociation of mant-ADP, which can be followed by the drop in mant-fluorescence that is associated with the displacement of mant-ADP from the nucleotide-binding pocket (see Supplementary Figure S3a and b). In the presence of Tpm4.2, the dissociation rate constant of ADP release from actin-NM-2A-S1 was reduced by approximately 13%, as shown in Fig. 1d (no Tpm: 6.05 ± 0.25 s^−1^; Tpm4.2: 5.29 ± 0.06 s^−1^; p < 0.01).

The intramolecular strain between the two heads of NM-2A-HMM is known to influence their ADP release rates^23^. The leading head that experiences a resisting intramolecular strain from the trailing head was shown to release ADP at a slower rate than the single-headed S1. The trailing head’s ADP release is stimulated by assisting strain. We determined the individual ADP release rates for the two HMM heads on bare and Tpm4.2-decorated actin filaments according to the protocol described by Kovacs *et al.*^23^. A preformed complex of actin-NM-2A-HMM with mant-ADP was rapidly mixed with a large excess of ADP (2 mM), which caused a drop in mant fluorescence upon mant-ADP displacement (see Supplementary Figure S3c and d). On bare actin filaments, the rate of ADP release from the leading head of HMM is 12.7-fold slower (0.48 ± 0.07 s^−1^; Fig. 1d) than the rate observed for the S1 construct. The trailing head releases ADP 1.5-fold faster (9.1 ± 2.2 s^−1^; Fig. 1d) than the S1 construct on bare actin filaments. Thereby, we confirm that our NM-2A-HMM construct exhibits strain-sensitive ADP release. A similar extent of gating was observed for the HMM construct in the presence of Tpm4.2 (0.50 ± 0.07 s^−1^; 7.5 ± 2.0 s^−1^; Fig. 1d).

### Tpm4.2 increases the number of actin landing events and the maximum filament sliding velocity on an NM-2A-coated surface

**Figure 2:**
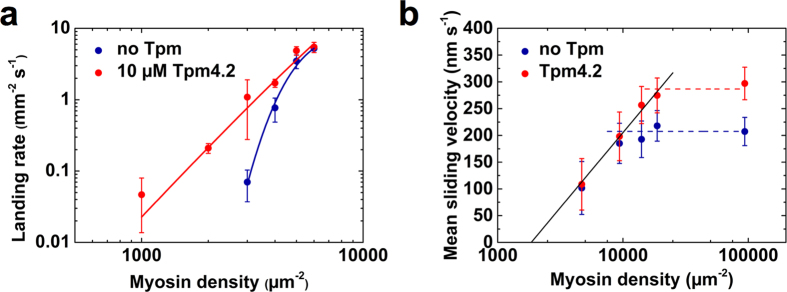
Tpm4.2 increases the number of actin landing events and the maximum sliding velocity on NM-2A-HMM-coated surfaces **(a)** Log-log plot visualising the relation between the landing rate and the surface density of NM-2A-HMM molecules. Landing assays were performed at 30 °C with bare actin or Tpm4.2-decorated filaments. Landing events were counted and averaged from 3 movies. Error bars indicate standard deviations. **(b)** Semi-logarithmic graph showing the relationship between the mean sliding velocity and the surface density of NM-2A-HMM molecules. Compared to bare actin filaments, Tpm4.2-decorated actin filaments display an extended range over which the increase in sliding velocity can be fitted with a linear slope. This leads to a 1.4-fold increase of the maximum sliding velocity for Tpm4.2-decorated filaments. *In vitro* motility assays were performed at 30 °C. The filament sliding velocities were recorded on three different areas of a flow cell. A Gaussian function was fitted to the velocity distributions. The points are mean sliding velocities from the Gaussian fits and the error bars indicate the standard deviations of the sliding velocities, which were also obtained from the Gaussian fits (see also Supplementary Figure S4).

We used landing assays to investigate the effect of Tpm4.2 on the interaction between NM-2A and actin filaments^29^. NM-2A-HMM was immobilised on the surface of a nitrocellulose-coated coverslip. In a TIRF microscope, we observed the transient interactions of fluorescently labelled actin filaments with the myosin-decorated surface. In the presence of 4 mM Mg^2+^-ATP, the landing rate of bare actin filaments dropped rapidly, when the NM-2A density on the surface was step-wise reduced. In contrast, the number of productive interactions with the surface dropped much slower when the actin filaments were decorated with Tpm4.2 (Fig. 2a). The data shown in Fig. 2a can be described with the model devised by Hancock and Howard^29^,


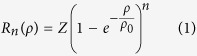


where R_n_ is the landing rate, Z is a parameter that determines the collision rate of filaments with the surface, ρ is the myosin density, ρ_0_ corresponds to the surface area over which myosins interact with an actin filament, and n can be interpreted as the minimal number of myosin molecules required for a productive landing event. The Tpm4.2-induced change of n in the landing assay from 43 ± 26 to 3.4 ± 1.5 shows that NM-2A is shifted towards strongly actin-attached states by Tpm4.2.

Next, we examined the influence of Tpm4.2 on the speed of translocation of actin filaments in an *in vitro* motility assay. We decorated coverslips with varying densities of NM-2A-HMM and recorded the movement of bare and Tpm4.2-decorated actin filaments following the addition of assay buffer containing 4 mM Mg^2+^-ATP. In the case of bare actin filaments the mean sliding velocity reached a plateau value of 203 ± 7 nm/s at a myosin density of approximately 10,000 molecules per μm^2^ (Fig. 2b; dashed blue line). The speed of Tpm4.2-decorated filaments continued to increase up to twice the surface density of NM-2A-HMM molecules, reaching a plateau value of 287 ± 11 nm/s (Fig. 2b; dashed red line). A widely accepted model by Uyeda *et al.*^30^,^31^ suggests that at high myosin densities the sliding velocity in the *in vitro* motility assay is limited by the detachment rate of myosin from actin, which in the case of NM-2A is determined by the rate of ADP release from the complex^18^. In the absence and presence of Tpm4.2, our stopped-flow experiments show no significant change in the rate of ADP release (Table 1). Therefore, the 1.4-fold Tpm4.2-mediated increase in velocity cannot be explained by a change in the rate of ADP release. It appears more likely that the Tpm4.2-mediated increase in velocity results from the improved coordination of the power-strokes of myosin heads interacting with an actin filament.

### NM-2A takes processive steps along actin filaments

**Figure 3:**
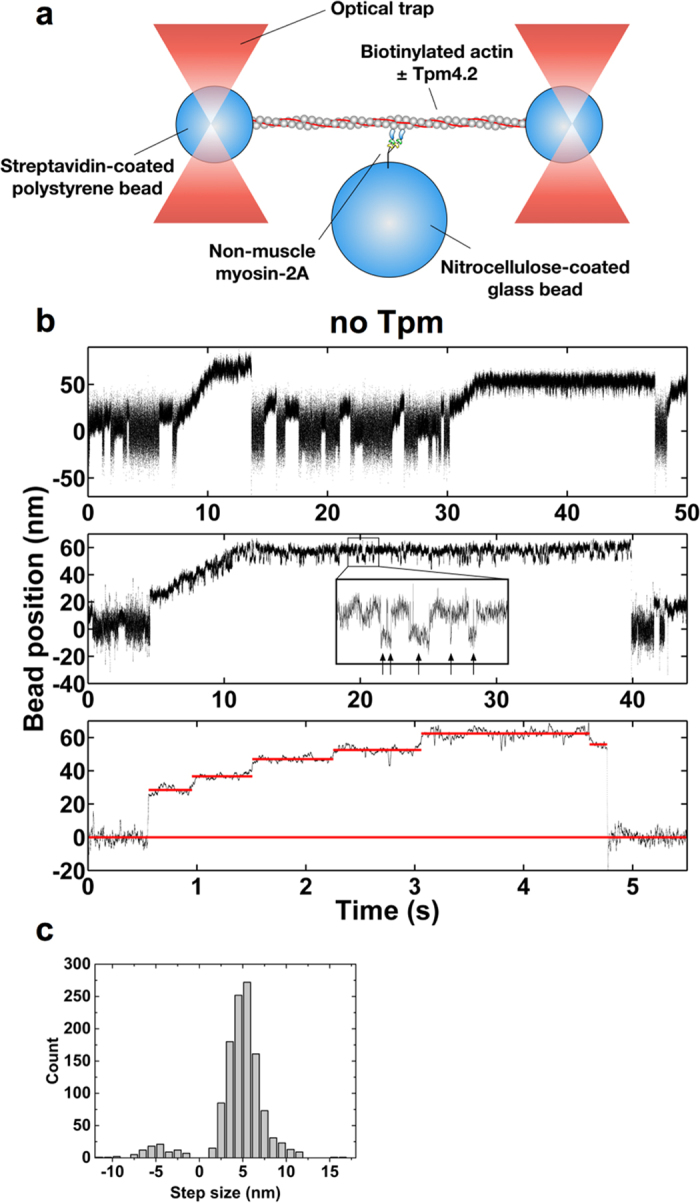
Two-headed NM-2A moves processively on F-actin **(a)** Schematic representation of the three-bead optical trap setup. **(b)** Examples of single NM-2A-HMM molecules interacting with single bare actin filaments at 2 mM ATP. The graphs illustrate movements of a trapped bead in direction of the actin filament axis. Top panel: Overview showing several binding events. Middle panel: Single processive run. Steps are visualised by applying a sliding mean filter (20 points average) to the data. The myosin moves a distance of approximately 50 nm. At an average resisting load of 3.4 ± 0.9 pN, it stalls by taking successive back- and forward steps. The insert shows five of the backward steps in detail (pointed out by arrows). Bottom panel: A single processive event with individual steps highlighted by red lines through the average bead position for each step. The bottom red line indicates the average free dumbbell position. The noise amplitude was reduced using a sliding mean filter (100 points average). **(c)** NM-2A-HMM follows the helical path of the actin filament stepping between neighbouring actin subunits. The step size on bare and Tpm4.2-decorated actin filaments shows no significant difference (see Supplementary Figure S5). Gaussian fits give a mean forward step size of 5.0 ± 1.6 nm and a mean backward step size of 4.7 ± 1.9 nm (n = 1204 steps; mixed from runs on bare and Tpm4.2-decorated filaments). The distance between neighbouring actin subunits corresponds to 5.5 nm^32^,^33^.

The rate of ADP release from the leading head of NM-2A-HMM is close to the overall cycle rate (see above), which suggests that the myosin is likely to take a step before both heads dissociate. NM-2A-HMM should therefore be able to perform short processive runs on actin filaments. We used a three-bead optical trap setup to observe single myosin molecules interacting with single actin filaments (Fig. 3a). Interactions between actin and NM-2A-HMM were detected by a reduction of the noise amplitude of the bead-actin-dumbbell position signal. Processive movement was detected by analysing the signal of the mean position along the filament axis of the dumbbell. In fact, we observed processive runs with NM-2A-HMM at 2 mM ATP on bare actin (Fig. 3b). While the majority of runs ended after a few steps, we frequently recorded runs in which the myosin moved longer distances up to a constant resisting force (stall force) of 3.4 ± 0.9 pN (number of events used to determine the stall force: n = 24). After reaching the stall force, the NM-2A-HMM construct underwent successive back- and forward stepping as illustrated in Fig. 3b.

Figure 3c illustrates the step size distribution of 1204 observed steps. Forward and backward step sizes appear as distinct populations. The apparent mean forward step size of NM-2A-HMM is 5.0 ± 1.6 nm. The apparent mean backward step size is 4.7 ± 1.9 nm. These values provide an estimate of the minimal step size, since the movement of the myosin is partially buffered by the compliance in the bead-actin link. We compared the measured step sizes at low displacement with those at high displacements, where the bead-actin link is strongly stretched and compliance is negligible (see Supplementary Figure S6). Considering this compliance effect, the mean values above underestimate the step sizes by approximately 10–20%. Thus, the measured step sizes are in good agreement with the distance of 5.5 nm between the neighbouring actin subunits in a strand^32^,^33^.

### Decoration of actin filaments with Tpm4.2 enhances the processivity of NM-2A in the presence of resisting loads

**Figure 4:**
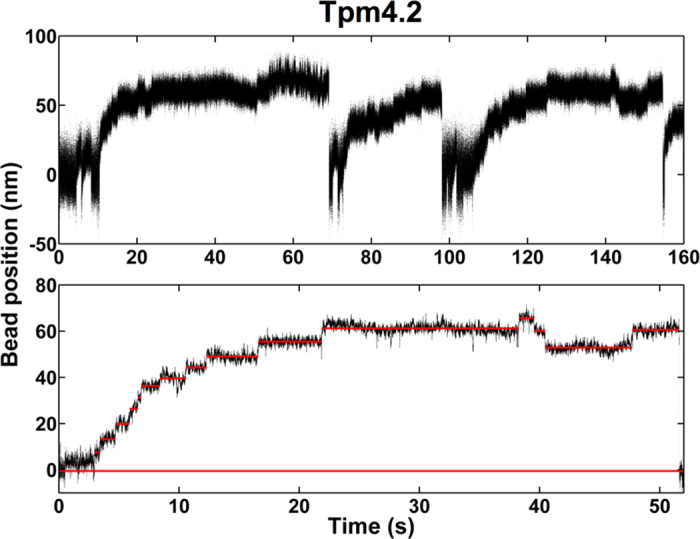
Tpm4.2 slows the stepping rate of NM-2A at high loads Examples of single NM-2A-HMM molecules interacting with single Tpm4.2-decorated actin filaments at 2 mM ATP.Upper panel: Overview showing three processive runs. Note that the slope of the events decreases successively, indicating prolonged periods between steps at high resisting loads. Lower panel: A single processive run of NM-2A-HMM on a Tpm4.2-decorated actin filament. Individual steps were visualised by applying a sliding mean filter to the data (100 points average). Red lines mark the average bead position between steps. The bottom red line highlights the mean free dumbbell position. Note the prolonged periods between steps at higher loads.

**Figure 5:**
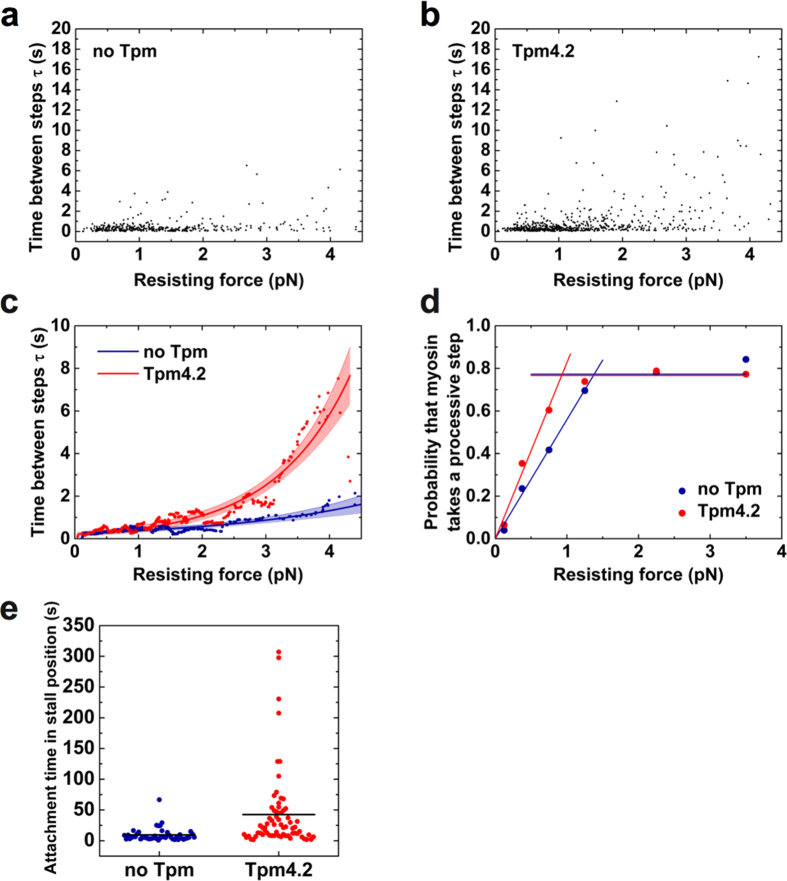
Load-dependent stepping and stall kinetics of NM-2A-HMM **(a)** Wait-times before NM-2A-HMM takes a step on bare F-actin, measured at different resisting loads acting on the myosin. Each data point represents the time interval between two steps (n = 371). The loads were calculated from the respective bead displacements. **(b)** Wait-times measured at different resisting loads before NM-2A-HMM takes a step on Tpm4.2-decorated F-actin (n = 580). **(c)** Tpm4.2 prolongs the wait-time before NM-2A-HMM takes a step under load. Equation (2) was fitted to the data in (**a**) (blue curve) and (**b**) (red curve). The curves represent best fits for the parameters k_step,0_ and δ. The coloured areas indicate 95% confidence limits based on the errors of the best-fit parameters (see text). A sliding mean filter (20 points window size) was applied to the data in (**a** and **b**) and plotted as blue and red points, respectively. **(d)** NM-2A-HMM shows load-enhanced processivity. Tpm4.2-decoration of the actin filament reduces the resisting force that is required to reach maximum processivity. The probabilities for a processive step were determined by relating the frequency of myosin taking a follow-on step (omitting initial steps) to the frequency of dissociation events. The frequencies were grouped into force bins (n = 19–182 frequencies per bin). Each bin centre is represented by a point in the diagram. The intersection of the two trend lines indicates the force at which the myosin processivity reaches its maximum. **(e)** Both the mean and maximum time NM-2A-HMM stays attached to actin in the stall position are increased 5-fold in the presence of Tpm4.2 (no Tpm: n = 45 events; Tpm4.2: n = 67 events). Horizontal lines indicate the mean attachment times.

On bare actin filaments, the stepping rate of NM-2A-HMM remains nearly constant during processive runs until the myosin reaches the stall plateau (Fig. 3b). In contrast, we observed a marked reduction in the stepping rate for myosin approaching the stall position on Tpm4.2-decorated actin filaments. As the examples shown in Fig. 4 illustrate, the wait-time between steps increased markedly at higher loads. Detailed comparison of the wait-times between steps recorded for runs on bare actin filaments and on Tpm4.2-decorated filaments (Fig. 5a and b, respectively) reveals that Tpm4.2 reduces the stepping rate of NM-2A when the myosin works under load. We applied a simple exponential model to describe the data shown in panels a and b of Fig. 5 (Fig. 5c).


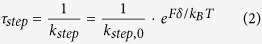


Here, k_step,0_ is the stepping rate at zero load, F is the resisting force, and δ is a distance parameter that is a measure of force-sensitivity of the stepping rate^34^,^35^,^36^,^37^. The thermal energy k_B_T was 4.07 pN·nm. According to this model, Tpm4.2-decoration has only a minor effect on the stepping rate of NM-2A at zero load (no Tpm: k_step,0_ = 3.94 (+1.72/−0.92) s^−1^; Tpm4.2: k_step,0_ = 4.95 (+3.54/−1.46) s^−1^). The stepping rate at zero load is similar to the ADP-release rates that we determined for the NM-2A-S1 and the trailing head of NM-2A-HMM in our stopped-flow experiments. This result suggests that the stepping rate is limited by ADP release. We did not determine the lifetimes of strongly-bound states that follow ADP release. However, others have shown that ATP-induced dissociation is an order of magnitude faster than ADP release for NM-2A at saturating ATP concentrations^18^. Therefore, it is unlikely that the rate of ATP-induced dissociation contributes to the stepping rate in our experimental setup. The distance parameter δ is increased 2-fold by the presence of Tpm4.2 (no Tpm: δ = 1.68 (+0.59/−0.35) nm; Tpm4.2: δ = 3.42 (+0.59/−0.44) nm). Based on these results, we conclude that Tpm4.2-decoration of actin filaments raises the energy barrier and changes the position of the force-sensitive transition state for taking a step, when the motor works against resisting loads. Extended dwell-times of the myosin under high loads are expected to support its processive behaviour. Therefore, we determined the probability of NM-2A-HMM taking a processive step as a function of the resisting force (Fig. 5d). Both, in the case of bare and Tpm4.2-decorated actin, we observed a steep rise in the probability of processive stepping with increasing loads. On bare actin filaments, the maximum probability for taking a processive step (0.77 ± 0.04) is reached at a resisting force of 1.38 ± 0.04 pN. In the presence of Tpm4.2, NM-2A reaches its maximum processivity (stepping probability of 0.77 ± 0.02) already at a resisting force of 0.93 ± 0.04 pN. The combination of both a slower stepping rate and increased processivity on Tpm4.2-decorated actin extends the attachment time for NM-2A working against an external force. As shown in Fig. 5e, both the mean and maximum time that NM-2A-HMM spent in the stall position until dissociation were prolonged 5-fold by Tpm4.2. Strikingly, we observed attachment times of more than 300 s on Tpm4.2-decorated filaments.

## Discussion

**Figure 6:**
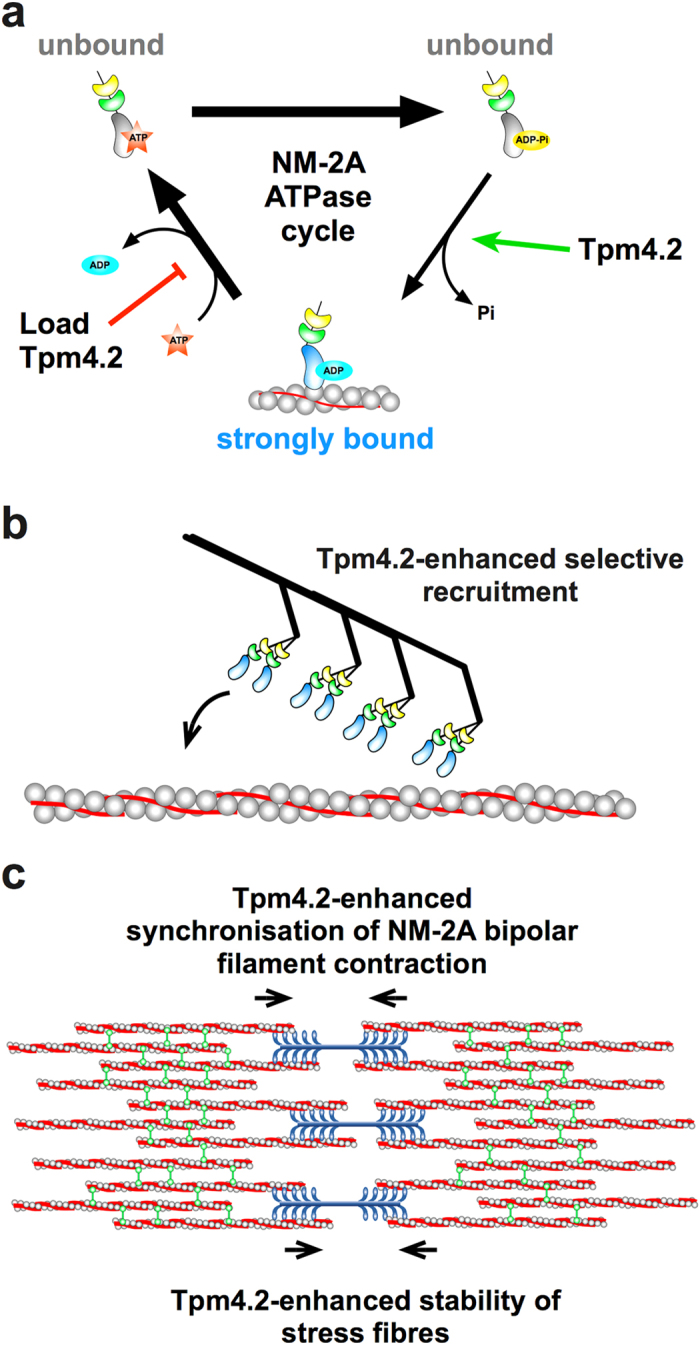
Model describing the modulation of NM-2A motor activity by Tpm4.2 and its implications for stress fibre function **(a)** Modulation of the NM-2A ATPase cycle by Tpm4.2. Tpm4.2 appears to affect the NM-2A ATPase cycle by accelerating the rate-limiting transition from the unbound myosin-ADP-P_i_ state to the strongly actin-bound myosin-ADP state. High resisting loads acting on NM-2A slow down its detachment. The detachment rate at high loads is further reduced by the presence of Tpm4.2, thereby increasing the effect of resisting loads on the duty-ratio of NM-2A. **(b)** Tpm4.2 provides a selectivity filter that enhances recruitment of NM-2A to a subpopulation of actin filaments. **(c)** Tpm4.2 supports NM-2A function in stress fibres by synchronising myosin heads and enhancing load-dependent processivity (blue: NM-2A, grey: actin, red: Tpm4.2, green: α-actinin).

We aimed to elucidate how the decoration of actin filaments with Tpm4.2 affects the motor properties of NM-2A. A cell-based study has previously demonstrated that Tpm4.2 binding to actin in emerging stress fibres recruits NM-2A to these filaments^11^. The cryo-EM structure of a Tpm-actin-myosin complex revealed stereospecific interactions between tropomyosin and myosin^38^. In the case of Tpm4.2-decorated actin filaments, the stereospecific contact area with NM-2A is predicted to be enlarged by approximately 300 Å^38^. Our kinetic analysis suggests that enhanced recruitment of NM-2A is achieved in part by accelerating the rate-limiting transition to the strongly actin-bound myosin-ADP state (Fig. 6a). Tighter binding and increased coupling between actin binding and release of the hydrolysis products can act like a selectivity filter for a specific myosin isoform. The avidity effect that results from NM-2A assembling into bipolar filaments may further enhance selective binding, leading to a situation were Tpm4.2-decorated actin filaments promote the specific recruitment of bipolar NM-2A filaments.

The kinetic properties of single-headed NM-2A-S1 were described to be compatible with a low duty-ratio motor^18^. However, experiments performed by the same group with NM-2A-HMM did already reveal a strain-sensitive ADP release step and gating between the two heads^23^. We confirm these results, as we find that ADP is released from NM-2A-HMM at two clearly distinct rates. The slow rate that is associated with ADP release from the leading head^23^ is slightly faster (0.48 ± 0.07 s^−1^) than the overall ATPase rate (0.22 ± 0.03 s^−1^), which is limited by re-binding of a detached head in the myosin-ADP-P_i_ state. These kinetic data predict that NM-2A should be able to take several processive steps under load-free conditions. Our optical trap experiments demonstrate that the extent of gating between the heads of NM-2A-HMM is indeed sufficient for the myosin to move in a processive fashion. As expected, most processive runs are short, caused by the frequent simultaneous dissociation of both heads. Different from myosins that are involved in the long-distance transport of cargo, such as myosin-5 and myosin-6, single molecules of NM-2A do not perform long distance runs. In their physiological environment multiple motors of NM-2A are assembled as bundles. Thus, sustained attachment to an actin filament is achieved by simultaneous binding of several motors of a bundle. Similar to NM-2B^36^, NM-2A takes short steps of 5.5 nm making it less suitable for long-distance transport, as this requires the motor to rotate around the actin filament axis. In contrast, a motor like myosin-5 takes 36 nm steps along the pseudo-helical actin filament repeat^39^, which allows the myosin to stay continuously on the same side of the filament. The fact that the processive stepping of NM-2A leads to a rotation of the interacting actin filament has interesting implications for stress fibre contractility. Katoh *et al.* have indeed provided evidence that actin filaments in stress fibres are rotated during contraction. The authors suggest that a rotation of actin filaments induces coiling of the stress fibres and thereby enables the fibres to shorten more extensively^40^.

The changes in the stepping rate observed in our optical trap experiments indicate that the detachment of NM-2A from F-actin is delayed in the presence of resisting forces. In the absence of Tpm4.2, the characteristic distance parameter δ (1.68 nm), which is a measure of load-sensitivity for the detachment rate, is low. The δ-value determined for NM-2A is similar to the δ-values reported for myosins that are involved in tension maintenance and work under high load conditions, such as smooth muscle myosin-2 (δ = 1.3 nm)^37^ and NM-2B (δ = 0.99 nm)^36^. In comparison, a δ-value of 4.3 nm was reported for a long-distance transporter like myosin-5^41^. The load-sensitivity of the detachment rate is amplified for NM-2A on Tpm4.2-decorated actin filaments with δ increasing 2-fold. Consequently, the detachment of NM-2A heads is strongly delayed under high loads. As a result, NM-2A will stay anchored to Tpm4.2-decorated filaments and move in a processive manner under high-load conditions.

In conclusion, our findings support a role of NM-2A as a motor that is involved in tension maintenance and contractility of cellular actin networks. The properties of Tpm4.2 are ideally suited to mediate the assembly of contractile actomyosin structures. We propose that Tpm4.2-decoration of actin filaments can act in part as a selectivity filter promoting the specific recruitment of NM-2A (Fig. 6b). The results from our *in vitro* motility assay provide evidence for the ability of Tpm4.2 to synchronise the power-stroke of NM-2A heads that are attached to the same filament. Once NM-2A is incorporated into an F-actin network and load is applied, the improved synchronisation of NM-2A molecules and their ability to step in a processive fashion will enhance the function of bipolar bundles and stabilise the contractile network (Fig. 6c). Studying the functional consequences of Tpm4.2-decoration of actin filaments for bipolar bundles of NM-2A in more detail is an interesting task for future experiments.

## Methods

### Steady-state ATPase measurements

NM-2A-HMM was phosphorylated at its regulatory light chain for activation of enzymatic activities. It was incubated for 30 min at 30 °C in ATPase Buffer (20 mM MOPS pH 7.0, 50 mM KCl, 2 mM MgCl_2_, 0.15 mM EGTA, 2 mM DTT) containing 1 mM CaCl_2_, 0.2 μM calmodulin (CaM), 2 μM essential light chain (MYL6), 2 μM regulatory light chain (MYL12b) and 46 nM myosin light chain kinase (MLCK). NM-2A-S1 was used without prior phosphorylation. The steady-state ATPase rates were determined in an NADH-coupled assay^42^ using 0.25 μM NM-2A-HMM or 0.1 μM NM-2A-S1 in ATPase buffer containing 2 mM ATP, 0.2 mM NADH, 0.5 mM phosphoenolpyruvate, 20 μg/ml lactate dehydrogenase and 50 μg/ml pyruvate kinase. Actin was pre-incubated for 30 min with Tpm4.2 at room temperature, before addition to the assay mix. Final assay concentrations were 0–60 μM actin and 0–15 μM Tpm4.2 for the NM-2A-HMM data. Since NM-2A-S1 has a lower actin affinity at steady-state than the HMM construct, 0–80 μM actin and 0–15 μM Tpm4.2 were used. The change in absorption at 340 nm was recorded in a 96-well plate on a microplate reader at 25 °C.

### ADP release kinetics

Transient kinetic experiments were performed at 20 °C using either a *HiTech Scientific SF-61DX2 or SF-61SX2* Stopped-Flow system (TgK Scientific Ltd, Bradford-on-Avon, U.K.). Tpm4.2-decorated actin filaments were formed by incubating actin filaments with 20 μM Tpm4.2 at room temperature for 30 min. The ADP-state complex of the S1 construct was formed by mixing 2 μM bare or Tpm4.2-decorated actin filaments, 2 μM NM-2A-S1 and 2 μM mant-ADP in MOPS Experimental Buffer (20 mM MOPS pH 7.0, 50 mM KCl, 5 mM MgCl_2_) containing 0–20 μM Tpm4.2. Displacement of mant-ADP was triggered by rapid mixing with an equal volume MOPS Experimental Buffer containing 4 mM ATP. The drop in mant-ADP fluorescence (365 nm excitation, 389 nm long-pass emission filter) upon dissociation from the myosin was recorded for 2 s. The fluorescence transients were best-described by fitting a single exponential decay function to the data.

The ADP-state complex of the HMM construct was formed by mixing 2 μM bare or Tpm4.2-decorated actin filaments, 0.4 μM NM-2A-HMM and 10 μM mant-ADP in MOPS Experimental Buffer containing 0 or 10 μM Tpm4.2. Displacement of mant-ADP was triggered by rapid mixing with an equal volume MOPS Experimental Buffer containing 2 mM ADP. The drop in mant-ADP fluorescence upon dissociation from the myosin was recorded for 20–50 s. The fluorescence transients were best-described by fitting a double exponential decay function to the data.

### Phosphate release kinetics

Phosphate release kinetics were measured as described earlier^18^,^43^. All tubing of the stopped-flow system was flushed with a phosphate mop of 0.5 units/l purine nucleoside phosphorylase and 0.4 μM 7-methylguanosine in ATPase Buffer and incubated for 1 h to remove free phosphate ions. All solutions were prepared in ATPase Buffer containing the phosphate mop. Before usage, 60 μM phalloidin-stabilized actin was mixed with 20 μM Tpm4.2 and incubated for 30 min.

By using the sequential mixing mode of the *HiTech Scientific SF-61DX2* Stopped-Flow system, 2.8 μM NM-2A-S1 and 2 μM ATP were mixed in a 1:1 ratio and incubated for 5 s. The generated myosin-ADP-P_i_ complex was then mixed 1:1 with actin or Tpm4.2-actin (30 μM post-mix concentration). All solutions contained 1.8 μM N-[2-(1-maleimidyl)ethyl]-7-(diethylamino)coumarin-3-carboxamide labelled phosphate binding protein (MDCC-PBP) as a phosphate sensor. The fluorophore was excited at 436 nm and the emitted light was passed through a 455 nm long pass filter. The fluorescence increase was described by fitting a single exponential function to the data.

### *In vitro* motility

*In vitro* motility assays were performed according to Kron and Spudich^44^. Flow cells were made by attaching a nitrocellulose-coated coverslip to a glass slide using two parallel strips of double-sided tape. Actin filaments were labelled with a molar excess of tetramethylrhodamine (TRITC) phalloidin overnight at 4 °C. Tpm4.2-decorated filaments were formed by pre-incubation of 10 nM labelled actin filaments for 30 min with 10 μM Tpm4.2 at room temperature.

For full activation of the NM-2A-HMM, RLC phosphorylation was carried out immediately prior to the experiments. The myosin was incubated for 30 min at 30 °C in Assay Buffer (20 mM imidazole pH 7.0, 50 mM KCl, 4 mM MgCl_2_, 0.15 mM EGTA, 2 mM DTT) containing 1 mM CaCl_2_, 0.2 μM CaM, 2 μM MYL6, 2 μM MYL12b and 92 nM MLCK.

Inactive myosins that could interfere with the assay were removed by spin-down with 5 μM actin in the presence of 2 mM ATP at 100,000 g in a table-top ultracentrifuge (*Beckman* Optima Max) at 4 °C. The supernatant was diluted in Assay Buffer to myosin concentrations of 25–500 μg/ml. Myosin densities on the nitrocellulose-surface were calculated assuming that all myosins in solution would completely bind to the surface. According to this assumption, a stock of 500 μg/mL NM-2A-HMM that is infused into the flow cell provides a density of 93,750 molecules per μm^2^. To reduce deviations in the myosin concentrations between the bare actin and Tpm4.2-actin cases as much as possible, the same myosin dilutions from the same myosin stock were used for the respective myosin densities. One chamber volume of the myosin dilution was infused into the flow cell. It was incubated for 2 min to allow the myosin to bind to the nitrocellulose-coated glass surface. The flow cell was then flushed with 3 volumes of BSA/AB (0.5 mg/ml bovine serum albumin in Assay Buffer) and incubated for 2 min to block the surface. Next, 10 nM TRITC phalloidin-labelled actin filaments in BSA/AB with 0 or 10 μM Tpm4.2 were infused and allowed to bind to the myosin surface for 2 min. Excess actin was washed out with 2 volumes of BSA/AB containing 0 or 10 μM Tpm4.2. Finally, BSA/AB containing 4 mM ATP, 0 or 10 μM Tpm4.2 and an anti-bleach system consisting of 5 mg/ml glucose, 0.1 mg/ml glucose oxidase and 0.02 mg/ml catalase was infused. Filament gliding over the myosin surface at 30 °C was observed on an *Olympus* IX70 Fluorescence Microscope with a 60 × Plan Apo 1.49 NA oil immersion TIRF objective and an additional 1.5-fold magnification. Filaments were excited with light of 550 nm wavelength in epi-fluorescence mode. Fluorescence light was filtered using a 580 nm band pass dichroic mirror and detected with an Orca Flash 4.0 CMOS camera (*Hamamatsu Photonics*). Filament movement was tracked using DiaTrack 3^45^.

### Landing assay

Decorated actin filaments were formed by pre-incubation for 30 min with 10 μM Tpm4.2 at room temperature. To assure a comparable filament length distribution, bare and Tpm4.2-decorated filaments were made from the same actin stock and treated similarly. NM-2A-HMM was prepared and myosin densities were calculated as described for the *in vitro* motility assay. First, the myosin was allowed to bind to the nitrocellulose for 5 min. Then, the surface was blocked with 3 volumes of BSA/AB for 2 min. Finally, 2 volumes of BSA/AB containing 4 mM ATP, 20 nM TRITC phalloidin-labelled actin filaments, 0 or 10 μM Tpm4.2 and anti-bleach solution containing 5 mg/ml glucose, 0.1 mg/ml glucose oxidase and 0.02 mg/ml catalase were infused. Filament landing rates were observed at 30 °C using the *Olympus* Fluorescence Microscope in TIRF mode and a 565 nm laser for excitation. Landing rates were counted manually from 30 s movies at a frame rate of 2 per second. Only filaments that touched the surface and were bound for at least two frames (1 second) were counted as an event. Filament lengths were measured using the *ImageJ* plugin *Jfilament*^46^. The average filament length was 4.1 ± 2.1 μm (mean ± SD) for bare filaments and 3.7 ± 2.3 μm for Tpm-4.2-decorated filaments. The difference in the landing rates is therefore not a consequence of differences in the filament length.

### Optical trap assay

Optical trap experiments were carried out as described previously (32,47 for setup). Flow cells were made as described for the *in vitro* motility assay, but the cover slips were coated with a suspension of 1.5 μm glass beads in 0.05% nitrocellulose in amyl acetate. Actin was coupled to polystyrene beads via biotin-neutravidin according to Ishijima *et al.*^48^. To visualise these biotin-coated 0.8 μM polystyrene beads (Kisker Biotech, Steinfurt, Germany) in the same fluorescence channel as F-actin, we treated them with a 1:100 mixture of Texas-Red-labelled streptavidin and unlabelled neutravidin. For Tpm4.2-decoration, TRITC phalloidin-labelled actin filaments were pre-incubated with 10 μM Tpm4.2 at room temperature for at least 15 min. NM-2A-HMM was phosphorylated as described for the *in vitro* motility assay, diluted to 0.5 μg/ml in Assay Buffer and infused into the flow cell. The myosin was allowed to bind to the surface for 1 min. Next, a blocking step with BSA/AB was carried out for 1 min. Finally, a mix of the streptavidin-coated polystyrene beads and 0.6 nM TRITC phalloidin-labelled biotinylated actin filaments in Assay Buffer containing 20 mM DTT, 1 μM phalloidin, 2 mM ATP, 0 or 10 μM Tpm4.2, 2 mg/ml glucose, 1 mg/ml glucose oxidase and 0.2 mg/ml catalase was added. The chamber was sealed with vacuum grease. The optical trap experiments were carried out on a custom-build system as described previously^47^. The trap stiffness was set to 0.06–0.08 pN/nm for both traps. The actin dumbbell was stretched to an initial bead-actin link stiffness of approximately 0.1 pN/nm. The link stiffness and its change during processive runs were considered when calculating resisting forces on the myosin. Motor activity was monitored by recording the position of the trapped beads using two 4-quadrant detectors at a sampling rate of 10 kHz. The difference in the noise amplitude between a myosin-bound and unbound actin dumbbell was increased by applying a low-amplitude (80 nm peak to peak), high-frequency oscillation (1 kHz sinusoid) to one of the traps^37^. The oscillation did not affect processive runs of the myosin. Data records were evaluated using MatLab routines. The traces of processive runs were tracked manually to find the transition between steps.

To ensure that observed processive events were the result of single NM-2A-HMM interactions, the glass bead surface was sparsely coated with the myosin construct. We assayed a flow cell with less than one activity on 15 platforms and still observed the same processive runs. For major data acquisition we worked in a regime where 51 out of 122 glass beads displayed ATP-sensitive interactions with the actin dumbbell. According to these statistics, the probability that an observed activity is due to multiple motors is approximately 29%. However, we observed processive movement with 44 of the 51 activities. The binomial probability that all 44 of these 51 activities are due to multiple motors is 2.7 × 10^−17^. This calculation does not take into account positional restraints on the bead surface that may limit the motors’ ability to interact with the same actin filament and to adopt a proper orientation to support processive movement. Considering these criteria, the probability that the events are caused by multiple motors is even lower. In addition, when applying a sliding mean filter to the data, individual steps of the myosin could be resolved (Figs 3b and 4a) and this clear staircase activity is unlikely to be the result of more than one motor.

Events that were caused by more than one motor could be easily recognised and sorted out as they usually produced multiples of the reported stall force and more than one stall plateau (see Supplementary Figure S7).

## Additional Information

**How to cite this article**: Hundt, N. *et al.* Load-dependent modulation of non-muscle myosin-2A function by tropomyosin 4.2. *Sci. Rep.* **6**, 20554; doi: 10.1038/srep20554 (2016).

## Supplementary Material

Supplementary Information

## Figures and Tables

**Table 1:** Summary of selected steady-state and transient kinetic parameters.

Parameter	Myosin construct	Method	No Tpm	Tpm4.2	Fold change^a^
k_cat_	NM-2A-HMM	NADH-coupled steady-state ATPase assay	0.22 ± 0.03 s^−1^	0.34 ± 0.02 s^−1^	1.55
K_actin_	NM-2A-HMM	NADH-coupled steady-state ATPase assay	30 ± 12 μM	29 ± 6 μM	–
k_cat_	NM-2A-S1	NADH-coupled steady-state ATPase assay	0.16 ± 0.05 s^−1^	0.23 ± 0.06 μM	1.44
K_actin_	NM-2A-S1	NADH-coupled steady-state ATPase assay	77 ± 57 μM	63 ± 55 μM	–
Phosphate release from actomyosin^b^	NM-2A-S1	Stopped-flow, MDCC-PBP	0.084 ± 0.003 s^−1^	0.179 ± 0.012 s^−1^	2.13
ADP release from actomyosin	NM-2A-S1	Stopped-flow, mant-ADP	6.05 ± 0.25 s^−1^	5.29 ± 0.06 s^−1^	0.87
ADP release from actomyosin	NM-2A-HMM	Stopped-flow, mant-ADP	c0.48 ± 0.07 s^−1^^d^ 9.1 ± 2.2 s^−1^	c0.50 ± 0.07 s^−1^^d^ 7.5 ± 2.0 s^−1^	–

Errors indicate: standard error from hyperbolic fit for k_cat_ and K_actin_; standard deviation for phosphate release rates (n = 5–7) and ADP release rates (n = 3–4).

^a^only significant changes (p < 0.05) are indicated.

^b^measured in the presence of 30 μM actin.

^c^leading head.

^d^trailing head.
