# Supplementary material for: Load-dependent modulation of non-muscle myosin-2A function by tropomyosin 4.2
Source: Sci Rep. 2016 Feb 5;6:20554. doi: 10.1038/srep20554 (PMC4742800; doi:10.1038/srep20554)
Supplement: Supplementary Information [file srep20554-s1.pdf]

Supplementary Information

Load-dependent modulation of non-muscle myosin-2A function by tropomyosin 4.2

Nikolas Hundt[1], Walter Steffen[2], Salma Pathan-Chhatbar[1], Manuel H. Taft[1], Dietmar J. Manstein[1,3,*]

[1]Institute for Biophysical Chemistry, Hannover Medical School, Carl-Neuberg-Str. 1, 30625 Hannover, Germany

[2]Molecular and Cell Physiology, Hannover Medical School, Carl-Neuberg-Str. 1, 30625 Hannover, Germany

[3]Research Division for Structural Analysis, Hannover Medical School, Carl-Neuberg-Str. 1, 30625 Hannover, Germany

*Correspondence to: manstein.dietmar@mh-hannover.de

Supplementary Figure S1

Coomassie-stained 12% SDS-PAGE showing the proteins used in this study. All proteins were produced at more than 90% purity. M: PageRuler Unstained Protein Ladder; 1: Human NM-2A-HMM. Heavy chain (157 kDa), regulatory light chain MYL12B (19 kDa), essential light chain MYL6 (17 kDa); 2: Human NM-2A-S1. Heavy chain (97 kDa), regulatory light chain MYL12B, essential light chain MYL6; 3: Chicken skeletal muscle α-actin (42 kDa); 4: Human Tpm4.2 (28 kDa)

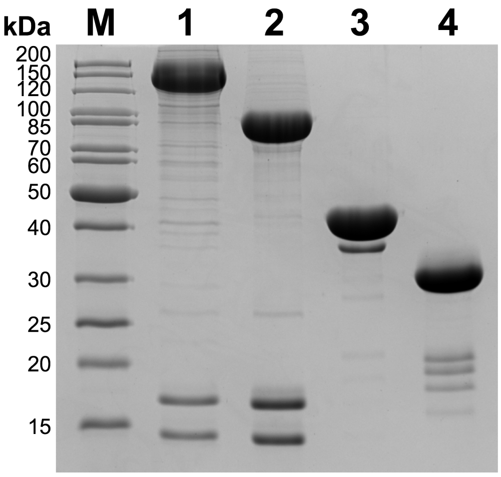

Supplementary Figure S2

The actin-activation of the NM-2A-S1 steady-state ATPase rate is increased by the presence of Tpm4.2. Error bars indicate the standard error of the mean. Data were described with a hyperbolic function. (n = 2 – 3)

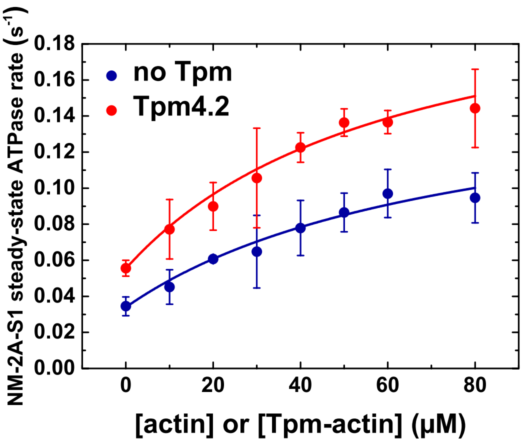

Supplementary Figure S3 (see also Figure 1D)

(a) Drop in mant-ADP fluorescence upon dissociation from the actin-NM-2A-S1 complex. **(b)** Drop in mant-ADP fluorescence upon dissociation from the Tpm4.2-actin-NM-2A-S1 complex. **(c)** Drop in mant-ADP fluorescence upon dissociation from the actin-NM-2A-HMM complex. **(d)** Drop in mant-ADP fluorescence upon dissociation from the Tpm4.2-actin-NM-2A-HMM complex. The data in (a) and (b) were described by single exponential decay functions. The data in (c) and (d) were described by double exponential decay functions. The fits are shown in red.

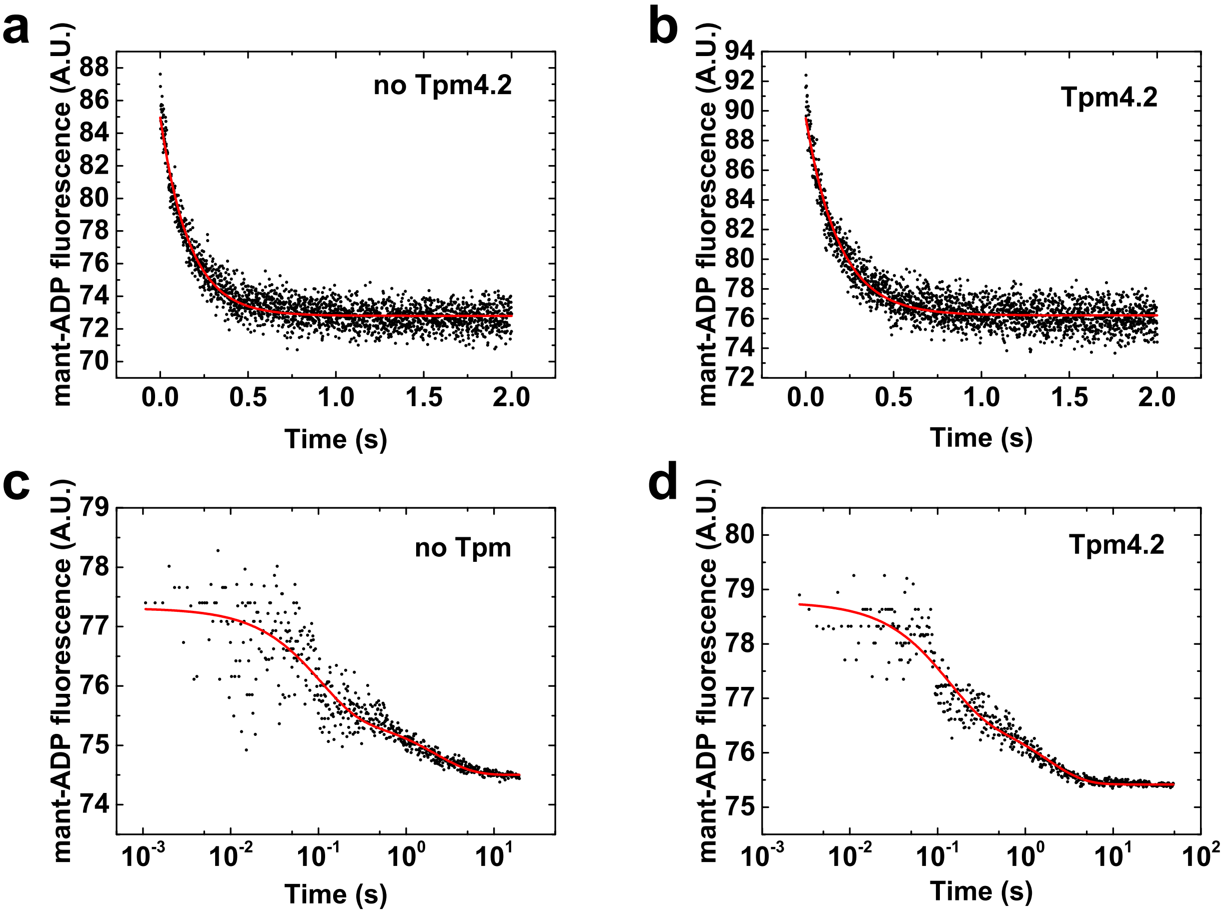

Supplementary Figure S4 (see also Figure 2b)

Filament sliding velocity distributions for flow cells that were coated with 500 µg/ml NM-2A-HMM. *In vitro* motility assays were performed at 30 °C. The points in Figure 2b are mean sliding velocities that were obtained from Gaussian fits to the distributions. The error bars represent the standard deviations of sliding velocities obtained from the same Gaussian fits.

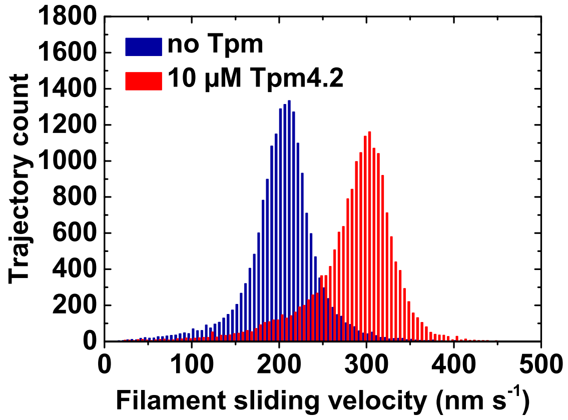

Supplementary Figure S5 (see also Figure 3c)

(a) Step size distribution of NM-2A-HMM running on bare actin filaments (n = 395 steps). **(b)** Step size distribution of NM-2A-HMM running on Tpm4.2-decorated actin filaments (n = 334 steps).

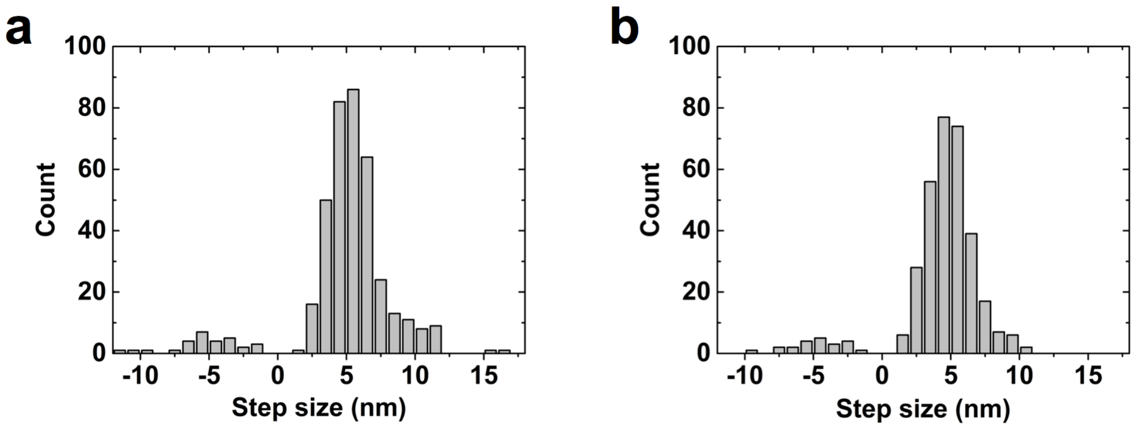

Supplementary Figure S6 (see also Figure 3c)

The step sizes shown in the histogram of Figure 3c were sorted for the respective displacements of the dumbbell. The red trend lines indicate an increase of the apparent step size at higher displacements. When the bead-actin link is stretched, the link stiffness increases and the movement of the myosin is less buffered by the bead-actin link compliance. Therefore, step size measurement at higher displacements better reflect the actual step size of the myosin. The trend lines show that the mean step sizes determined from the histogram in Figure 3c underestimate the step size of NM-2A by approximately 10 – 20%.

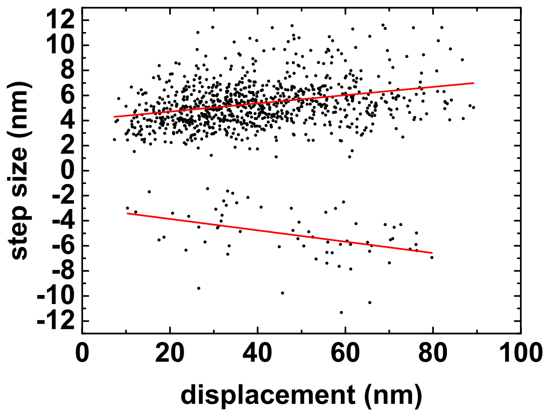

Supplementary Figure S7

Example of a processive run on a Tpm4.2-decorated actin filament that is caused by the action of two or more myosins. The dumbbell is clearly moved to more than one stall plateau and reaches multiples of the reported stall force. These multiple motor interactions were not taken into account for calculations.

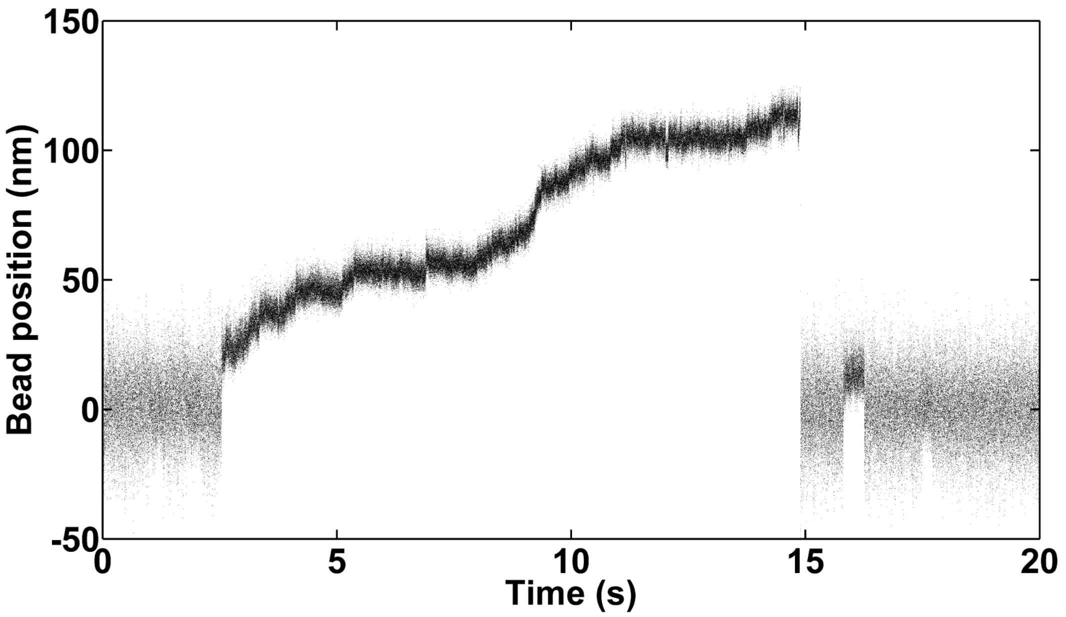

Supplementary Methods

Cloning and virus production

The cDNA encoding human Tpm4.2 was purchased from SourceBioscience. The coding sequence was amplified in a PCR using primers *Tpm4.2-forward* (5' TAT ACC ATA TGG CCG GCC TCA ACT CCC 3') and *Tpm4.2-reverse* (5' AGC TCC TCG AGT TAT ATA

CAG TTA AGT TCG TTT AGT G 3‘) and cloned into the NdeI and XhoI sites of pET-23a(+).

For the NM-2A-HMM construct, the DNA sequence encoding amino acids 1-1337 was amplified from the full-length cDNA by PCR using primers *NM-2A-HMM-forward* (5’ CGC GCG GCG CGC ATG GCA CAG CAA GCT GCC GAT A 3’) and *NM-2A-HMM-reverse* (5’ CAG CTG GTC GAC GTG GTG ATG ATGATGATGATGATG CTC GTC CTC CAC CTG CTT GAG CTT 3’). Using restriction endonucleases BssHI and SalI, the amplicon was inserted into the polyhedrin site of a pFastBac1 vector with an Avi-tag encoding sequence. The product was used for the over-expression of NM-2A-HMM with C-terminal His_8- and Avi-tags for purification and optional biotinylation.

The DNA of a NM-2A-S1 construct (amino acids 1-837) with C-terminal His_8-tag was amplified using primers *NM-2A-HMM-forward* and *NM-2A-S1 (KpnI)-reverse* (5’ CCA TGG GGT ACC TTA GTG GTG ATG ATG ATG ATG ATG ATG ATG CAG CGG CTT GAC CTT GGT GGA G 3’). The amplicon was inserted between the BssHI and KpnI sites of a pFastBac1 vector without the Avi-tag encoding sequence.

The cDNAs of human non-muscle essential light chain (*MYL6*) and regulatory light chain (*MYL12b*) were purchased from SourceBioscience. *MYL12b* was amplified using primers *MYL12b forward* (5’ GAG CTC CTC GAG ATG TCG AGC AAA AAG GCA AAG A 3’) and *MYL12b reverse* (5’ CCA TGG GGT ACC TCA GTC ATC TTT GTC TTT GGC TCC AT 3’). *MYL6* was amplified using primers *MYL6-forward* (5‘ CCTAGG GGATCC ATG TGT GAC TTC ACC GAA GAC CA 3‘) and *MYL6-reverse* (5‘ TTCGAA AAGCTT TCACCCCGACAGGATATGCCT 3‘). The *MYL12b* cDNA was inserted between the XhoI and KpnI sites of the p10 promoter site and the MYL6 cDNA between the BamHI and HindIII of the polyhedrin promoter site of a pFastBacDual vector.

Bacmids were produced by transformation of *E. coli DH10Bac* with the pFastBac vectors according to the manufacturer’s instructions. Bacmids were transfected into Sf9 (*Spodoptera frugiperda*) cells and virus-amplification was performed as described by the manufacturer.

Protein expression and purification

Human Tpm4.2 was produced in *E. coli* Rosetta(DE3)pLysS by transformation with the pET-23a(+) vector containing the Tpm4.2 cDNA. Cells were grown at 37 °C to an OD of 0.6 and induced with 1 mM IPTG. Expression was carried out at 37 °C for 3-4 hours. Cell pellets were suspended in Tpm4.2 Lysis Buffer (20 mM imidazole pH 7.0, 50 mM NaCl, 1 mM $MgCl_2$, 2 mM DTT, Protease Inhibitor Cocktail) and disrupted by sonication on ice. Genomic

DNA was degraded by addition of DNase I and 10 min incubation on ice. Cell debris was spun down at 100,000 *g* for 1 h at 4 °C. The supernatant was filtered through a 1.2 µm filter. It was heated to 80 °C for 10 min, then slowly cooled down to 4 °C again. This step results in precipitation of all proteins except for Tpm4.2. The precipitate was removed by centrifugation at 20,000 *g* for 20 min at 4 °C. To reduce nucleic acid contamination Tpm4.2 was twice precipitated at pH 4.6. The pellet was resuspended in Anion Exchange Low Salt Buffer (20 mM imidazole pH 7.0, 50 mM NaCl, 1 mM DTT) and subsequently loaded onto a DEAE Sepharose column. It was eluted with a linear gradient of Anion Exchange High Salt Buffer (20 mM imidazole pH 7.0, 500 mM NaCl, 1 mM DTT). Tpm4.2 was completely separated from the remaining nucleic acids. Finally, to enrich full-length species, Tpm4.2 was purified by size exclusion chromatography on a HiLoad 16/60 Superdex 75 column. Remaining degraded species were less than 10% (see gel in Fig. S1). The purified protein was concentrated, flash-frozen in liquid nitrogen and stored at –80 °C.

For NM-2A-HMM or S1 production Sf9 cells were co-infected with baculovirus encoding for the respective heavy chain and virus encoding for both light chains MYL6 and MYL12b. For each construct optimal virus dilutions and expression periods were determined empirically by SDS-PAGE and immune-blot analysis. Both constructs were purified in a similar way. The cell pellets were suspended in NM-2A Lysis Buffer (50 mM HEPES pH 7.4, 300 mM NaCl, 4 mM $MgCl_2$, 0.3 mM EDTA, 0.3 mM EGTA, 30 mM imidazole, 2 mM ATP, 1 mM DTT, Protease Inhibitors) and disrupted by sonication. Cell debris was removed by centrifugation for 45 min at 100,000 *g* at 4 °C. The supernatant was incubated for 1.5 hours with Ni-NTA superflow. The affinity matrix was washed with NM-2A Lysis Buffer and with NM-2A Wash Buffer (25 mM HEPES pH 7.4, 300 mM NaCl, 4 mM $MgCl_2$, 70 mM imidazole, 1 mM DTT). Finally, the protein was eluted with NM-2A Elution Buffer (25 mM HEPES pH 7.4, 200 mM NaCl, 2 mM $MgCl_2$, 250 mM imidazole, 1 mM DTT, 2 µM each MYL6 and MYL12b). The S1 construct was only dialyzed against Myosin-2A Storage Buffer (25 mM HEPES pH 7.4, 200 mM NaCl, 2 mM $MgCl_2$, 0.1 mM EGTA, 1 mM EDTA, 1 mM DTT) and then prepared for storage. The HMM was subsequently purified by size exclusion chromatography on a HiLoad 16/60 Superdex 200 column. The proteins were concentrated, supplemented with 30% sucrose, flash-frozen in liquid nitrogen and stored at –80 °C. Both constructs can be stored at –80 °C without loss of activity for several months. Once thawed, the constructs were kept on ice and used for experiments the same day.

Chicken skeletal muscle actin was purified from chicken breast using the method of Lehrer and Kerwar[1].

Supplementary References

1. Lehrer, S. S. & Kerwar, G. Intrinsic Fluorescence of Actin. *Biochemistry* **11,** 1211–1217 (1972).
